# Extraction of Peppermint Essential Oils and Lipophilic Compounds: Assessment of Process Kinetics and Environmental Impacts with Multiple Techniques

**DOI:** 10.3390/molecules26102879

**Published:** 2021-05-13

**Authors:** Aleksandar Radivojac, Oskar Bera, Zoran Zeković, Nemanja Teslić, Živan Mrkonjić, Danijela Bursać Kovačević, Predrag Putnik, Branimir Pavlić

**Affiliations:** 1 Faculty of Technology, University of Novi Sad, Blvd. Cara Lazara 1, 21000 Novi Sad, Serbia; aradivojac@yahoo.com (A.R.); obera@uns.ac.rs (O.B.); zzekovic@tf.uns.ac.rs (Z.Z.); zivan_mrkonjic@hotmail.com (Ž.M.); 2 Emergent BioSolutions, 5901 East Lombard St, Baltimore, MD 21224, USA; 3 Institute of Food Technology, University of Novi Sad, Blvd. Cara Lazara 1, 21000 Novi Sad, Serbia; nemanja.teslic@fins.uns.ac.rs; 4 Faculty of Food Technology and Biotechnology, University of Zagreb, Pierottijeva 6, 10000 Zagreb, Croatia; dbursac@pbf.hr; 5 Department of Food Technology, University North, Trg Dr. Žarka Dolinara 1, 48000 Koprivnica, Croatia

**Keywords:** *Mentha piperita* L., hydrodistillation, microwave-assisted hydrodistillation, supercritical fluid extraction, extraction kinetics modeling, essential oil

## Abstract

Consumers are becoming more mindful of their well-being. Increasing awareness of the many beneficial properties of peppermint essential oil (EO) has significantly increased product sales in recent years. Hydrodistillation (HD), a proven conventional method, and a possible alternative in the form of microwave-assisted hydrodistillation (MWHD) have been used to isolate peppermint EO. Standard Soxhlet and alternatively supercritical fluid (SFE), microwave-assisted, and ultrasound-assisted extraction separated the lipid extracts. The distillations employed various power settings, and the EO yield varied from 0.15 to 0.80%. The estimated environmental impact in terms of electricity consumption and CO_2_ emissions suggested that MWHD is an energy efficient way to reduce CO_2_ emissions. Different extraction methods and solvent properties affected the lipid extract yield, which ranged from 2.55 to 5.36%. According to the corresponding values of statistical parameters, empiric mathematical models were successfully applied to model the kinetics of MWHD and SFE processes.

## 1. Introduction

Peppermint (*Mentha piperita* L.) is a plant from Lamiaceae family that is cultivated worldwide. Peppermint leaves and their essential oil (EO) have therapeutic properties as a gastric stimulant and carminative. Peppermint oil is among the most valued and extensively used EOs in flavoring of medications and formulations for oral care, chewing gums, cough syrups, sweets, and beverages. Peppermint EO has been found to have antioxidant, antiviral, antibacterial, antifungal, and antiparasitic activities [1].

Peppermint leaves have approximately 1.2–3.9% (*v*/*w*) of EO with more than 300 compounds detected. The main constituents are menthol and its derivatives (menthone, isomenthone, menthyl acetate, etc.) and eucalyptol. The chemical composition of peppermint leaves and oils differs with plant maturity, variety, geographical area, and growing environment [2]. Organic production is a practice of sustainable cultivation based on ecological principles through the rational use of natural resources, the consumption of renewable energy sources, the preservation of biodiversity and the protection of the ecosystem.

Consumers tend to choose organically grown products that are free of insecticides, herbicides, artificial stimulants, and other chemicals commonly used in agriculture. The “back-to-nature” trend has created a new markets for high-priced organic products EO. The global demand for mint EO is expected to reach USD 330.2 million by 2025 [3].

Hydrodistillation (HD) and organic solvent extraction are the old-style methods commonly used for EO extraction. These traditional methods have several drawbacks in terms of the quality of the extracted EOs. The main concerns associated with traditional approach are the potential degradation of sensitive compounds and the presence of organic solvent residues in the EO/extract [4,5]. Moreover, such extraction procedures are labor-intensive. Advanced extraction technologies, e.g., microwave-assisted hydrodistillation (MWHD), microwave-assisted extraction (MAE), supercritical fluid extraction (SFE), and ultrasound-assisted extraction (UAE) have emerged as solutions to these drawbacks of traditional methods.

Microwave-assisted methods for EO isolation have been developed as desirable processes for use at laboratory and industrial scales. The application of microwave as an energetic vector is defined by the property of materials to absorb a portion of the electromagnetic energy and convert it into thermal energy. In contrast, conventional heating is based on conduction and convection, while part of the heat could be lost. Microwave heating depends on direct electromagnetic interactions with polar solvents/materials. These interactions are mainly due to two main phenomena occurring simultaneously: ion conduction and dipole rotation [6]. Dipole molecules tend to align themselves with the electric field to be in the appropriate phase. Due to the constant change in the direction of the electric wave the molecules will constantly try to realign themselves, producing kinetic energy and frictional force from collision of adjacent molecules which generates heat. Although dried plant material is typically used for isolation of EO, plant cells still contain some amount of moisture, which serves as a target for microwave heating. The rapid temperature increases cause an internal pressure rise [7]. This leads to the ruptures in the cellular structures and rapid EO diffusion into the medium.

Microwave-assisted hydrodistillation (MWHD) is based on microwave heating in which the EO released from the plant material is carried away by water vapor. Microwave-assisted extraction (MAE) is another technique where microwaves are applied for acceleration of EO extraction where a greater extraction rate can be achieved along with reduced process costs. The focused microwave heating the cell walls to rupture, allowing EO to flow into the surrounding organic solvent [8].

Supercritical fluid extraction (SFE) is an excellent alternative to conventional extraction with organic solvents which has been established as a green and environmentally friendly method. Fluids at their critical temperature and pressure exhibit altered physiochemical properties that differ from those of gasses or liquids under standard conditions. The physiochemical properties of supercritical fluids include density, viscosity, diffusivity, and dielectric constants, which are simply controlled by the process parameters. 

Low critical temperature (31.1 °C) and pressure (73.8 bar) are the main reason why carbon dioxide is suitable for the extraction of sensitive molecules. The exceptionally low surface tension and high diffusion of supercritical CO_2_ allow easy penetration into the sample and dissolution of the desired components. The dissolving power of supercritical CO_2_ is suitable for the selective recovery of non-polar to slightly polar compounds from the EO. The extracted material can be effortlessly recovered by simply releasing the pressure which allows evaporation of the solvent from the extract [9].

Ultrasound-assisted extraction (UAE) is considered an efficient, cost-effective, and simple technique. Extraction enhancement by ultrasound has been attributed to various physical and chemical phenomena. The main mechanisms of ultrasonic extraction are based on acoustic cavitation [5]. Acoustic cavitation stands for the formation, expansion, and implosive collapse of bubbles that occur when ultrasonic waves propagate in liquid media. Numerous physical effects occur when air bubbles implode near or against the surface of a sample. High velocity jets and shock waves propel the liquid toward the plant surface, causing localized erosion and fragmentation. The reduced particle size, and resulting increased surface area combined with macroturbulence, micro-mixing and interparticle collisions at high velocity enhance mass transfer. The ultrasonic capillary effect is responsible for the improved solvent penetration. The rapid rehydration and swelling of a plant material has a positive effect on the basic extraction mechanisms [10,11].

In this research, conventional HD and alternative MWHD were applied for the isolation of pure EO from organically grown *M. piperita*. Traditional Soxhlet and novel SFE, MAE and UAE techniques were applied for the isolation of lipophilic compounds. The influence of the applied method and various parameters on the overall extraction yield was studied. In addition, mathematical modeling of MWHD and SFE processes was used to obtain valuable information for larger scale process implementation.

## 2. Results and Discussion

### 2.1. Particle Size of Extraction Materials

To ensure intensification of mass transfer, the plant material should be reduced to a suitable particle size, within relatively narrow limits, before the extraction step. The particle size of grounded leaves ranged from 100 to 2 mm [7] which was large-enough surface area for plant matrix and the solvent to improve the extraction efficiency. The tissue structure of the ground plant material is disrupted, while a smaller particle reduces the duration of solvent diffusion and improves the mass transfer rate from the solid to the liquid phase. Additionally, finer particles allow much deeper and improved microwave penetration as well [12]. If the particle size is sufficiently small in the extraction, the majority of cells will be ruptured by the application of ultrasound and internal diffusion becomes less essential phase [13].

**Figure 1:**
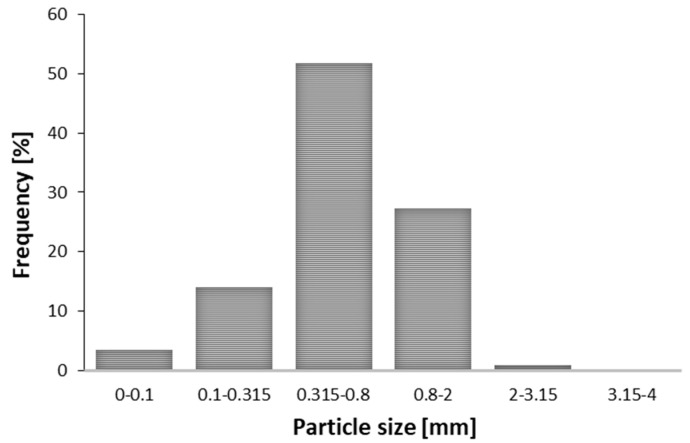
Particle size distribution in peppermint sample.

The plant material in our experiments was properly prepared, with only 1% of the particles above the defined upper limit (2 mm). The fine particle fraction, with a diameter of less than 0.315 mm, represented 17% of the sample. Finally, the mean particle size of the prepared *M. piperita* sample was 0.4 mm. The moisture content in the *M. piperita* sample was 8.65%. It is desirable that the plant material contains some amount of water. The water in the plant material heats up, evaporates, and increases the internal pressure, which leads to cell disruption and hence better extraction yield. Concerning SFE, the moisture content of the dry peppermint leaves had negligible influence on the extraction yield [14]. Particle size distribution of peppermint samples was presented in Figure 1.

The main limitation with application of smaller particles is the complexity of matrix separation from the liquid phase after the extraction. Generally, filtration or centrifugation is used for separation, and the exploitation of fine particles can be technical challenge [6]. In the case of SFE, the problem of channel formation within the extraction bed can occur if the particles are too small. Formed channels alter the solvent flow and prevent contact between the solvent and the plant material, which can result in lower yield and process efficiency. In addition, the production of fine particles by grinding may cause the loss of volatile compounds [15]. According to European Pharmacopoeia [16], the preferred fraction of the plant material passes through a sieve with an opening of 2 mm. Additionally, as a technical recommendation, the proportion of fine particles with a diameter of less than 0.5 mm should not exceed 10%.

### 2.2. Separation of EO

**Figure 2:**
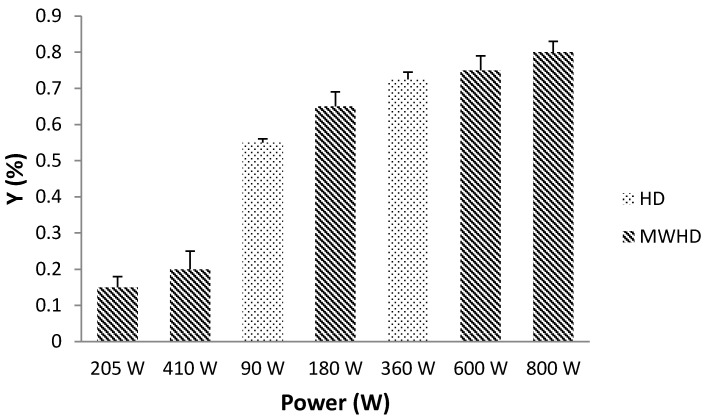
The effect of applied power in HD and MWHD on total extraction yield of EO.

HD is conventional and the most extensively used method for extracting EOs from the aromatic plants and medicinal herbs. Accordingly, this conventional method is a great reference point for EO extraction. A comparison of the EO extraction yields between HD and MWHD is shown on Figure 2.

The total hydrodistillation yield (Y_EO_) of EO, obtained from peppermint was 0.73% by HD (410 W) and 0.80% by MWHD with 800 W irradiation power. In both cases, Y_EO_ increased with the increase of applied power. Microwave irradiation showed a major increase in Y_EO_. In the case of HD, EOs are recovered by conventional heating of a mixture of water and plant material, followed by liquefaction of the vapors in the condenser [17]. MWHD induces swift distribution of energy over the entire volume of solvent/sample, causing instant surge in temperature. Influence of microwave power and temperature is proportionally interrelated hence the temperature will rise rapidly with the higher radiation power. Furthermore, the viscosity and surface tension of the solvent are reduced at elevated temperatures, which improves solvent penetration and soaking of the plant material. Selective heating of the sample matrix causes a sudden rise in temperature and pressure inside the cells, which triggers the rupture of the compact cell wall in the sample. Consequently, the EO is rapidly exposed to the surrounding medium. The higher microwave extraction potential is probably due to the combined effects of heat and mass transfer phenomena acting in the same direction.

### 2.3. Separation of Lipid Extract

Soxhlet extraction, which is “golden” standard for gauging the efficiency of numerous alternative extraction procedures, gave the highest Y_E_, 3.86 and 5.36% with *n*-hexane and methylene chloride, respectively. However, this lengthy extraction consumed considerable time and heat. The use of large amounts of harmful organic solvents is another significant drawback. Heat-reflux extraction is based on a consecutive permeation and solubilization steps that enhance the diffusion of analytes from the sample.

A high Y_E_ was achieved by UAE, in 40 min, 75% (UAE-Hex), and 99% (UAE-MeCl) of the extract was recovered, while MAE required 30 min to achieve only about 60% extraction efficiency. The mechanism of MAE exposes the target compounds to the solvent caused by the cellular disruption. In the case of UAE, the various physical and chemical effects stimulated by the ultrasound enhanced the permeability of the plant tissue, thus facilitating the release of the cells contents [10]. The total amount of extracted compounds is strongly associated with the solvent polarity. The higher dielectric constant of methylene chloride (ε = 8.93) explains a considerably better yield. This is particularly important in the case of MAE, where it is clearly specified that only solvents with a permanent dipole are heated under microwave. Solvents with low dielectric constant, such as hexane (ε = 1.89) are transparent to microwaves, hence no thermal energy is released with its exposure to radiation. When microwave transparent solvents are used, selective heating of the sample matrix is mainly responsible for the extraction mechanism. This approach can be remarkably functional for the extraction of thermosensitive components to avoid their degradation [6].

Since SFE is a high-pressure technology, it could be concluded that pressure is the most important process parameter that has shown a positive influence on the total Y_E_. Increasing the pressure can lead to better matrix penetration, which enables higher extraction efficiency. More importantly, pressure is related to density, which has a positive effect on the solubilizing power of the supercritical fluid. Increasing the pressure (100, 200, 300 and 400 bar) has effect of increasing the fluid density (628.7, 839.9, 910.0, and 956.1 kg/m^3^, respectively), which improves Y_E_. Rise in pressure from 100 to 200 bar resulted in a significant increase in solvent density, leading to an increase in Y_E_ from 2.62 to 3.52%. However, further increase in pressure did not provide any significant rise of the solvent density and the absence of any significant difference in Y_E_ (≈3.6%) can be observed at higher pressure level.

**Figure 3:**
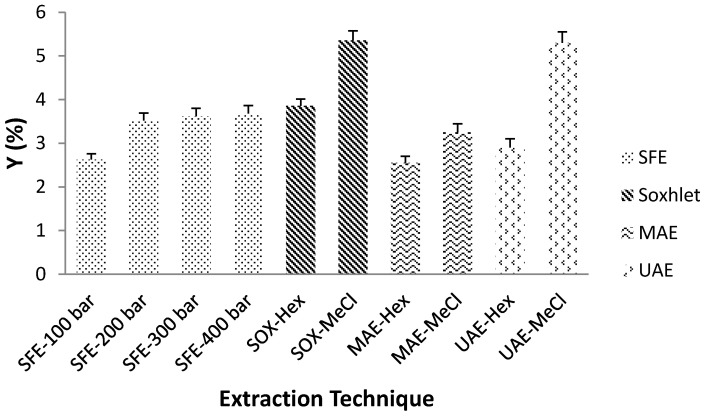
The effects of applied extraction technique and process conditions on total extraction yield (Hex—hexane and MeCl—methylene chloride).

SFE boosts solubility of fluids above their critical point. However, regardless of the extraction mechanism, all extraction methods are based on appropriate solvent selection. Appropriate choice of solvent ensures a more effective extraction process. The choice of solvent mainly depends on reaching high affinity among the supercritical CO_2_ and the target molecules, penetration of the solvent, and its interaction with extraction material. Ideally, the solvent will be highly selective for target components and exclude undesirable and concomitant molecules. Compatibility of the solvent with following analytical steps is another critical aspect [6]. Moreover, different physical properties of the solvent need to be considered while selecting a suitable solvent for novel extraction methods. The selection of solvent for MAE is determined by the solvent’s ability to absorb the microwave energy and use it for heating, which is generally high for polar solvents with high dielectric constant and a high dielectric loss. The dielectric constant (ε) determines the degree of the absorption, while the dielectric loss represents the measure of the matrix ability to absorb microwave energy and consequently release it as a heat to nearby compounds determining the efficiency of microwave irradiation. Solvent viscosity is another important parameter that alters the dipole rotation and thus the ability of the solvent to generate heat. In the case of UAE, the physical properties of the solvent, such as viscosity, surface tension, and vapor pressure, must be considered. These physical parameters control the occurrence of acoustic cavitation and more specifically the cavitation threshold [18]. An increase in viscosity or surface tension causes an improvement in the cohesive forces between the solvent molecules and thus a significant increase in the cavitation threshold. A solvent with low vapor pressure is generally preferred due to the effective collapse of cavitation bubbles comparing to solvents with high vapor pressure [19]. Theoretically, any fluid can be used as a solvent in supercritical state, but the desirable properties of these solvents should be low toxicity, cost, and high solvent selectivity towards target molecules. Due to its critical temperature and pressure, low cost, wide availability, high purity, non-flammability, and ecological safety (GRAS), CO_2_ is the most suitable supercritical solvent [20]. Extraction yield (Y_E_) for different extraction techniques and solvents is depicted in Figure 3.

### 2.4. Kinetics Modeling of HD and MWHD

The goal of engineering is to operate the process at optimal conditions. Optimal conditions are usually understood to be the condition of the plant system that allows the realization of maximum yield, reduced operating costs and time, in short, the economic optimum. Process modeling serves as a useful tool for its design and optimization at either laboratory or industrial level. The kinetics of HD and MWHD were fitted by different and commonly used mathematical models while statistical parameters for goodness of fit (*R*^2^ and AARD) were listed in Table 1. The text continues here (Figure 2 and Table 1).

A mean coefficient of determination (*R*^2^) ranging from 0.971 to 0.981 combined with tolerable AARD suggested the satisfactory fit of experimental data. This indicates that all four empirical models can be applied to predict process performances for both HD and MWHD. When it comes to MWHD at 90 W, the *R*^2^ was overly low for all applied models. This is rather expected due to the lack of energy provided by the microwave oven in distinct emission periods, as power level dictates the working period for magnetron. At 90 W, the magnetron switched-on-cycle is rather short which does not ensure a continuous heating and distillation. The largest mean coefficient of determination (*R*^2^ = 0.981) and the lowest AARD (7.27%) show that the model with simultaneous washing and diffusion (Model I) provides the best fit. In contrast, the second-order model (Model IV) provided the poorest fit of the experimental results. The model II, which assumes immediate washing followed by diffusion, and the model III (diffusion without washing step) provided almost identical fitting quality. Kinetic models can contribute to the fundamental understanding of the phenomena which occur in distillation process. To comprehend which phenomena control the distillation process, the values of the model parameters must be considered (Table 2).

Judging by Model I, EO from the surface of broken cells is available for rapid washing and distillation, while EO from unbroken cells must initially diffuse slowly to the surface. The value of the Model I parameters *k_d_*_1_ and *k*_1_ increased with the power for both HD and MWHD. At lower power levels, it can be stated that washing phase occurs rapidly comparing to the internal diffusion since the washing rate constant *k*_1_ was significantly higher. The values of *k_d_*_1_ and *k*_1_ were practically identical when higher power levels were applied. Statistical parameters imply that the appropriateness of the model improved with the number of variable parameters. However, the parameter *f* (EO fraction due to washing) had a negative value. It can be concluded that Model I does not describe the distillation process with complete accuracy. Model II suggests that the rapid washing phase occurs instantly at the beginning of the distillation, and then a slow diffusion phase takes place. The effects of the heating method and power levels in the case of parameter *f* were rather complex. For both HD and MWHD, parameter *k_d_*_1_ improved as power increased. This was also noticed for the Model III. The second-order rate constant *k_d_*_2_ from Model IV increased with the increase of distillation power. However, the influence of the microwave energy input on this constant was not clear. Although the empirical models might not completely account for the phenomena governing distillation process, they still could be used to predict the equilibrium EO yield (*q_∞_*). Model II and Model III proposed highly comparable equilibrium EO yields with the experimentally determined ones. At higher power levels Model I also provided a good agreement with the experimental data. Lastly, Model IV predicted a much higher equilibrium EO yield. Parameter analysis indicated that Model III, based on the pseudo-first kinetics, credibly described the distillation process.

**Figure 4:**
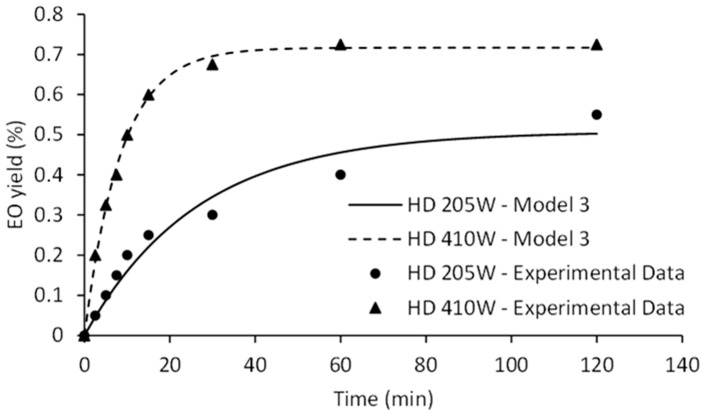
The effect of HD power on kinetics of EO isolation.

Fitting of experimental data and influence of the power input on HD kinetic is illustrated in Figure 4.

**Figure 5:**
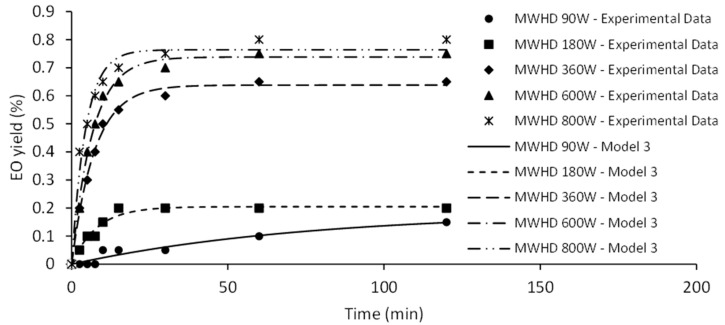
The effect of microwave irradiation power on MWHD kinetics.

Model III represents a diffusional model based on material balance across internal surface of plant cell assuming that the components to be extracted are homogeneously distributed within the plant cell and the surface resistance is insignificant [21]. The first step of the curve is linear, corresponding to the diffusion rate constant (*k_d_*_1_). Second step of the curve approaches the limit value determined by equilibrium amount of EO (*q_∞_*). The EO yield during the initial phase of HD was significantly improved at 410 W. Surge in diffusion rate constant may be credited to the increased availability of EO. Certainly, increase of the heating power of HD causes the rapid disrupture of the plant matrix. Similarly, higher positive slopes (*k_d_*_1_) were observed as the microwave irradiation power increased (Figure 5).

Microwave energy acts as a driving force that enables the disintegration of the sample and leakage of EO to its surface [22]. Therefore, an amplified irradiation power will enhance rate of the distillation and shorten time required for equilibrium yield. Hence, higher MWHD irradiation power (600 and 800 W) has a positive effect on the extraction kinetics and can generally achieve higher yields compared to the HD, similar to previously reported findings [23].

When evaluating a new extraction technology, it is important to also evaluate the impact on the environment. Coal, oil, and natural gas are currently the world’s most important sources of energy. The use of these fossil fuels increased quantities of greenhouse gases in the atmosphere. Carbon dioxide, a major greenhouse gas, has a dominant influence on global warming, climate change, and ozone layer depletion. It follows that the increased concentration of CO_2_ in the atmosphere has a harmful effect on human society and the global economy. Therefore, energy efficiency has essential role in the perspective of sustainable development, as it enables energy saving and reduction of CO_2_ emissions. Estimated electrical consumption and CO_2_ emission of HD and MWHD is shown in Table 3.

**Figure 6:**
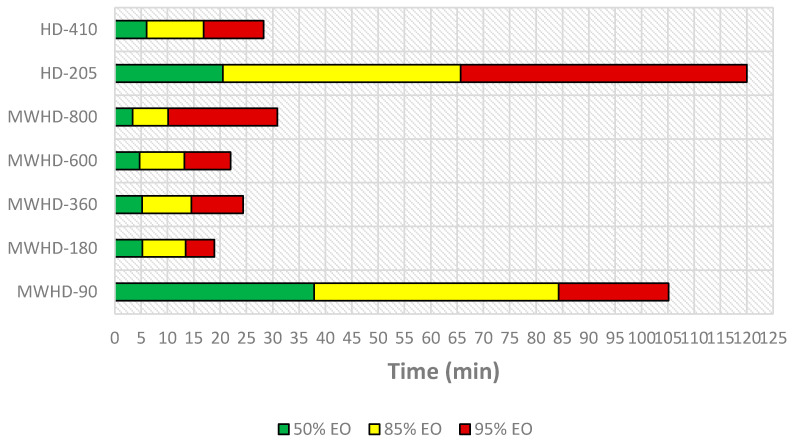
Timeline of the EO distillation process.

In order to optimize and operate an energy efficient process, many factors have to be analyzed. From an economic perspective, the most important aspect is to find the right balance between the cost base and the value of the extracted EO. Figure 6 illustrates timeline of distillation process.

**Figure 7:**
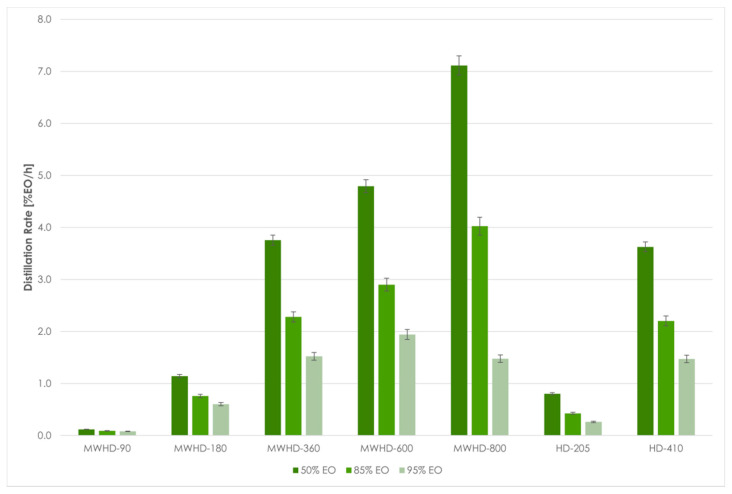
MWHD and HD distillation rate at different thresholds.

**Figure 8:**
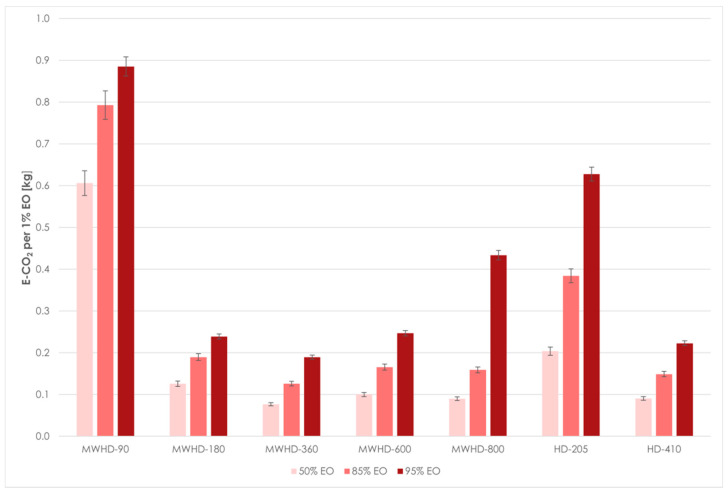
Environmental impact of MWHD and HD at different thresholds.

From the results obtained using Model III follows that the extraction time required to reach certain distillation thresholds differed between the applied techniques and power levels. Even at first glance, the HD-205 and MWHD-90 can be considered wasteful. In other samples, the extraction times were noticeably short. The time required to extract 50% of the available EO was 3.37, 4.69, 5.19, and 5.24 min for MWHD at 800, 600, 360, and 180 W, respectively, while it was 6.04 min for HD-410. MWHD was evidently faster than the traditional HD where longer extraction times improved the yield. However, this benefit seems to be exceedingly diminished with prolonged extraction. Accordingly, another 6–11 min are required to reach the next extraction threshold (85% EO). After that, it takes 5–21 min to extract only 10% of the extractable EO. Thus, with longer extraction time, the distillation rate decreases drastically (Figure 7). Moreover, the power consumption increases considerably. Therefore, prolonging the process has a negative environmental impact (Figure 8) and is generally not economically viable.

The comparison of distillation rates and CO_2_ emissions shows that the heating duration is an important factor to be investigated. If the extraction duration is appropriately optimized, MWHD offers an energy-efficient way to improve extraction yields and reduce CO_2_ emissions. Moreover, shortening the extraction time could be beneficial to avoid possible thermal degradation and oxidation of sensitive target compounds.

### 2.5. Kinetics Modeling of SFE

The application of mathematical models enables the evaluation of the extraction process and additional exploitation of the experimental results. Mathematical equations applied for description of SFE kinetics are combination of mass-transfer based models, empirical models, and models based on heat-transfer analogy [24]. Two empirical models were applied for fitting the SFE of *M. piperita* samples. Furthermore, pressure influence on kinetic curves and adjustable model parameters at fixed temperature (40 °C) and CO_2_ flow rate (0.3 kg/h) were evaluated. The same statistical parameters (*R*^2^ and AARD) were used for determination of accordance between experimental results and applied empirical models (Table 4).

**Figure 9:**
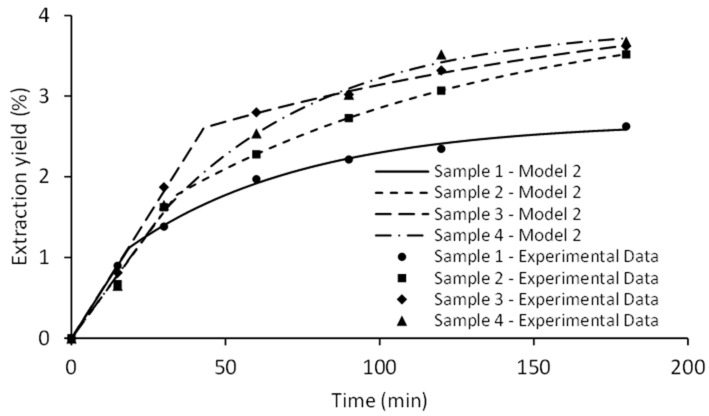
The effect of pressure on SFE kinetics fitted by Sovová model.

Remarkably high values of *R*^2^ and low AARD indicate a satisfactory fit in the case of both applied models, with the Model II being slightly better (Figure 9). Calculated model parameters are presented in Table 5.

*Y_∞_* and *k* were the variable parameters in Model I. The pressure exhibited a positive effect on the parameter *Y_∞_*, which represents the asymptotic total extraction yield. Elevated pressure increased CO_2_ density, which is associated with an enhanced solvating power of CO_2_. Operating the SFE under higher pressure is not always recommended, as decreased extraction selectivity is likely to dilute the content of target compounds within the extract [25]. There was no clear trend for the influence of pressure on the parameter *k*, since high values were observed at 100 and 300 bar (0.0258 and 0.0220 min^−1^), and a decrease at 200 and 400 bar (0.0166 and 0.0168 min^−1^). The Model II has already been successfully implemented to model the SFE process [26,27]. This model is characterized by five variable parameters, most of which correlate with several phenomena that affect mass transfer in SFE [28]. A graphical representation of the Model II is shown in Figure 9.

The first part of the curve corresponds to the cumulative extract recovered in the rapid extraction phase, which is recognized as the constant-extraction rate period (CER). CER period determined with parameter *t*_1_ was shorter than 45 min in all cases, implying that the initial extraction phase is solubility-controlled due to excellent transport properties of supercritical CO_2_. Calculation of SFE kinetic parameters during this extraction phase could be significant for scaling the process to the industrial level because the prolongation of the process following the CER period is usually not economically justified [29]. Subsequent extraction step relates to the internal diffusion-controlled phase. This falling extraction rate (FER) period was characterized by the parameter *t_i_*, which fluctuated from 55.31 to 157.96 min. Extraction kinetic during the FER period might be relevant only if target compounds are extracted after the CER [30]. Mass-related partition coefficient (*K_m_*) varied between the low values for SFE-200, SFE-300, and SFE-400 (≈0.16) and the maximum value obtained at the minimum pressure (0.2696 at 100 bar). All SFE runs were performed with a uniform raw material, minimizing its impact on the variable parameter associated with the degree of particle fragmentation and cell rupture (G). However, different pressure levels considerably influenced parameter *G*. The highest value (0.5971) was noticed at 300 bar. The peak values of the parameters *t*_1_ and *t_i_* were also observed in the same SFE run. Furthermore, the FER phase was not completed after 180 min, indicating that the total extraction time should be prolonged in order to completely exhaust the plant material at 300 bar. However, the FER period is less relevant for the majority of the SFE industrial processes. In the case of SFE-400, a relatively long *t*_1_ was achieved compared to the experimentally applied extraction time due to the notably high percentage of extractable compounds during the CER period. Limitation of the applied empirical models in predicting the extraction yield should be highlighted as the asymptotic yield (*Y_∞_*) provided significantly higher values compared to the experimentally obtained results.

## 3. Materials and Methods

### 3.1. Plant Material and Chemicals

This Plant material was organically cultivated peppermint which was kindly donated by the Institute of Field and Vegetable Crops (Novi Sad, Serbia) in 2015. The peppermint leaves (*M. piperitae folium*) were dried at room temperature, properly stored and kept at ambient temperature prior to further use. Leaves were ground in a blender and average particle size same as particle size distribution were analyzed with set of sieves (CISA Cedaceria Industrial, Spain). Water content of ground and dried peppermint leaves was gravimetrically analyzed by drying the plant material at 105 °C in laboratory oven (Sterimaric ST-11, Instrumentaria, Zagreb, Croatia) until constant weight. All experimental trials were performed in three replicates and results were presented as average value ± standard deviation. Carbon dioxide (99.9%) was purchased from Messer Tehnogas AD., Novi Sad, Serbia. *n*-hexane was purchased from Merck KgaA, Darmstadt, Germany, and methylene chloride was obtained from Centrohem, Stara Pazova, Serbia. All other chemicals and solvents used for extraction and separation were of analytical reagent grade.

### 3.2. Isolation of Essential Oil

#### 3.2.1. Conventional Hydrodistillation

The essential oil content (EO) in plant material was analyzed according to the slightly adjusted official method [16,23]. Briefly, 40.0 g of dried and ground peppermint leaves was transferred in a laboratory glass balloon (1 L) and filled with 400 mL of distilled water. Hydrodistillation (HD) was performed in a glass Unger apparatus for 120 min. In order to evaluate HD kinetics, yield (Y) of EO was measured after 2.5, 5, 7.5, 10, 15, 30, 45, 60, 90, and 120 min of process and presented as % *v*/*w*. The kinetics of HD was analyzed for two levels of the irradiation power (205 and 410 W).

#### 3.2.2. Microwave-Assisted Hydrodistillation

Microwave-assisted hydrodistillation (MWHD) was performed with microwave oven (MM817ASM, Bosch, Germany) with adjusted glass apparatus according to the procedure described elsewhere [23]. Similarly to HD, 40.0 g of plant material was transferred in a laboratory glass balloon (1 L), filled with 400 mL of distilled water, and placed in oven for the MWHD. Extractions were performed at five power levels of the heater (90, 180, 360, 600, and 800 W) for total of 120 min with measurement of EO yield at the same time periods as HD. The mixture of water and EO was evaporated through glass pipe connector and collected in Unger apparatus after the condensation. The Y of EO was presented as % (*v*/*w*).

### 3.3. Isolation of Lipophilic Compounds

#### 3.3.1. Soxhlet Extraction

Ten grams of plant material was extracted in Soxhlet apparatus individually by two organic solvents (methylene chloride and *n*-hexane, 120 mL of each). Extraction was performed with at least 15 exchanges of extract which lasted ~6 h. After the extraction, the residual solvent was removed under vacuum and obtained peppermint extract was further dried at 40 °C for 24 h. Solvent-free extracts (SOX-HEX and SOX-MeCl) were transferred in glass vials stored at −18 °C prior to further use.

#### 3.3.2. Ultrasound-Assisted Extraction

For ultrasound-assisted extraction (UAE), 20.0 g of plant material was mixed with 200 mL of solvent (*n*-hexane—UAE-HEX, and methylene chloride—UAE-MeCl) in 500 mL glass flasks and placed in sonication bath (EUP540A, Euinstruments, France). Fixed extraction parameters were: temperature (50 °C), ultrasonic power (60 W/L), frequency (40 Hz), and extraction time (40 min). Glass flask was connected to the condenser in order to prevent loss of solvent due to evaporation. Peppermint extracts were filtered under vacuum through filter paper immediately after extraction, collected in glass vials, and kept at −18 °C prior to further use.

#### 3.3.3. Microwave-Assisted Extraction

Equipment setup adjusted for microwave-assisted extraction (MAE) of bioactive compounds from peppermint leaves was previously reported by Zeković et al. [31]. Briefly, 20.0 g of plant material was mixed with 200 mL of extraction solvent (*n*-hexane—MAE-HEX or methylene chloride MAE-MeCl) in 500 mL glass flask. Flask with solvent and plant material was placed in a microwave oven (MM817ASM, Bosch, Germany) and connected to the glass condenser through a hole at the top of oven to prevent evaporation of solvent. MAE were performed at fixed microwave irradiation power (360 W) and fixed extraction time (30 min). Obtained extracts were immediately filtered under vacuum after MAE and concentrated with a rotary evaporator at 40 °C. Lipid extract were then put in glass vials and stored at −18 °C prior to further use.

#### 3.3.4. Supercritical Fluid Extraction

Extraction of peppermint with supercritical CO_2_ was conducted on laboratory scale extraction plant (HPEP, NOVA-Swiss, Effretikon, Switzerland) with features thoroughly described by Pekić et al. [32]. For each supercritical fluid extraction (SFE) 70.0 ± 0.01 g of plant material was placed in extractor. All experimental runs were conducted at fixed temperature (40 °C), CO_2_ flow rate (0.3 kg/h) and extraction time (180 min), while pressure was varied for each SFE (100, 200, 300, and 400 bar). After SFE, extracts were separated from solvent under conditions set at 15 bar and 25 °C. Solvent-free peppermint extracts were collected in glass vials and kept at −18 °C prior analysis.

#### 3.3.5. EO and Total Extraction Yield

The extraction yield of EO and lipid extract achieved by the conventional and novel extraction and distillation techniques was obtained by:

(1) $$Y\;\left[\%\right]=\frac{\mathrm{volume}\;\mathrm{of}\;\mathrm{essential}\;\mathrm{oil}\;\mathrm{or}\;\mathrm{mass}\;\mathrm{of}\;\mathrm{lipid}\;\mathrm{extract}}{\mathrm{mass}\;\mathrm{of}\;\mathrm{peppermint}\;\mathrm{sample}}\times 100$$

Results were expressed as percentage (%), i.e., *v*/*m* and *m*/*m* for EO and lipid extract, respectively. 

### 3.4. Kinetics Modeling

#### 3.4.1. Distillation Kinetics

Assumptions as the foundation for distillation kinetics modeling were described in details by Milojević et al. [33]. Briefly:

- water–plant material mixture in the distillation flask is perfectly mixed;
- the EO is considered as a single component;
- plant material particles are considered as isotropic, equal in size, shape, and initial EO content;
- the effective coefficient of diffusion through plant particles is constant;
- resistance of the EO mass transfer from the external surfaces of the plant particles could be neglected;
- the EO and hydrolate are completely immiscible;
- a fraction of the EO is located at the external surfaces of the plant particles (ƒ), and the rest is uniformly distributed in the plant particles (1 − ƒ);
- the isolation of EO occurs via two simultaneous mechanisms: (a) “washing” of the EO from the external surfaces of the plant particles and (b) the diffusion of EO from the interior of the plant particles towards their external surfaces.

Four empirical models based on these assumptions were used for modeling of hydrodistillation kinetics and model equations were given in Table 6:

#### 3.4.2. SFE Kinetics Modeling

SFE curves (4 experimental trials) were fitted to models obtained from empirical equations commonly used for kinetics modeling of similar processes. Empirical equation of the first model is determined by a specific case of Fick’s law, which could be modified with substitution of adjustable parameter *Y_∞_* with the initial content of the solute in the solid phase (*x*_0_) [36]:

(2) $$\mathrm{Model}\;I:\;Y=Y_{\infty }\left(1-e^{-kt}\right)$$

where *Y* stands for total extraction yield (%); *Y_∞_* is total yield obtained for infinite time of extraction process (%), adjustable parameter specific for each set of process parameters (as all adjustable parameters); *k* represents rate constant (min^−1^); *t* is extraction time (min).

The second model equation was derived from simplified mathematical SFE process models [28]. Aforementioned models are determined by characteristic times during SFE, i.e., time of mass transfer in the fluid phase (*t_f_*), time of internal mass transfer (*t_i_*), time of extraction equilibrium (*t_eq_*), and mean residence time of CO_2_ in the extractor (*t_r_*). For modeling SFE kinetics which includes a plug flow in the extractor, Sovová suggested the following equations:

(3) $$\mathrm{Model}\;\mathrm{II}:\;Y=Y_{\infty }G\frac{t}{t_{1}}\;\mathrm{for}\;t\leq t_{1}=\frac{G}{K_{m}\dot{q}}$$

(4) $$Y=Y_{\infty }\left(1-\left(1-G\right)e^{-\left(\frac{t-t_{1}}{t_{i}}\right)}\right)\;\mathrm{for}\;t\geq t_{1}$$

where *G* represents particle size and fragmentation; *K_m_* stands for mass-related coefficient and represents the equilibrium of the mass concentration on particle surface; and $\dot{q}$ is a specific solvent flow rate (kg CO_2_/kg plant h). *G*, *K_m_*, and *t_i_* are adjustable model parameters. 

### 3.5. Environmental Impact of EO Distillation

Electrical consumption and CO_2_ emission were calculated to provide insights about environmental impact. The electrical consumption (*A*) of HD and MWHD were calculated as the electrical power for a time, using the following equation [19]:

(5) A=P×t

where *A* is electrical consumption (kWh), *P* is electrical power (kW), and *t* is time (h).

CO_2_ emission was calculated by the equation:

(6) $$E_{CO_{2}}=\frac{A\times 800}{1000}$$

where *E_CO*_2_*_* is CO_2_ emission (kg) and *A* is electric consumption (kWh) since 800 g of CO_2_ will be rejected in the atmosphere during the combustion of fossil fuel to obtain 1 kWh from coal or fuel [20].

### 3.6. Statistical Analysis

The goodness of fit and ability of applied mathematical models to describe the experimental data were determined by the statistical parameters such as average absolute relative deviation (AARD) and the coefficient of determination (*R*^2^).

## 4. Conclusions

It is anticipated in the future that rising consumer preferences for products based on organic and natural ingredients will support demand for essential oils derived from aromatic plants. Increasing production demand, international energy crises, and rising costs are the major reasons for the development of energy preservation methods. By using MWHD, the yield of EO could be improved rapidly and significantly. Increasing the MWHD power generally improves the EO yield. However, higher microwave power might alter the chemical content of EO on account of the potential (thermal) destruction of certain molecules. Another notable difference is the lower relative energy consumption and reduced CO_2_ emission. Advanced extraction techniques, UAE and MAE, have significantly reduced the time and organic solvent consumption compared to traditional procedure, i.e., Soxhlet extraction. However, the extraction yield was not significantly improved. SFE performance is highly dependent on the pressure parameter, which controls the solvent properties. The SFE also extracts the oil in less time than the Soxhlet method. The kinetic models were successfully used to describe the MWHD and SFE processes. Although the mathematical models do not fully elucidate the phenomena that dominate the extraction processes, they can still be applied to improve the extraction process and reduce the operating time and cost.

## Figures and Tables

**Table 1:** Statistical parameters for goodness of fit between experimental and modeled data.

Run	Model I		Model II		Model III		Model IV	
	*R* ^2^	AARD (%)	*R* ^2^	AARD (%)	*R* ^2^	AARD (%)	*R* ^2^	AARD (%)
HD								
205 W	0.996	7.71	0.964	13.82	0.961	13.21	0.980	8.23
410 W	0.997	2.88	0.997	2.64	0.997	2.88	0.990	5.14
MWHD								
90 W	0.932	20.15	0.928	23.10	0.928	22.41	0.929	21.57
180 W	0.975	6.81	0.969	7.03	0.969	7.55	0.937	12.72
360 W	0.994	3.69	0.994	3.58	0.994	3.69	0.986	5.48
600 W	0.995	3.96	0.995	3.75	0.995	3.96	0.981	7.62
800 W	0.980	5.68	0.980	5.21	0.980	5.68	0.998	1.75
Mean	0.981	7.27	0.975	8.45	0.975	8.48	0.971	8.93

**Table 2:** Calculated parameters of four empirical models applied for HD and MWHD kinetics modeling.

Run	Experiment	Model I				Model II			Model III		Model IV	
	*q* *_∞_*	*q* *_∞_*	*k_d_*_1_	*k*_1_	*ƒ*	*q* *_∞_*	*k_d_*_1_	*ƒ*	*q* *_∞_*	*k_d_*_1_	*q* *_∞_*	*k*_2_
HD												
205 W	0.55	1.99	0.0016	0.1086	0.880	0.53	0.0297	0.060	0.51	0.0379	0.63	0.0603
410 W	0.73	0.72	0.1175	0.1175	−0.275	0.72	0.1156	0.011	0.72	0.1175	0.80	0.1884
MWHD												
90 W	0.15	0.33	0.0043	0.0574	0.906	0.18	0.0140	−0.008	0.19	0.0132	0.28	0.0332
180 W	0.20	0.20	0.0664	0.0741	−6.033	0.20	0.1286	−0.022	0.21	0.1252	0.23	0.7161
360 W	0.65	0.64	0.1366	0.1366	−0.001	0.64	0.1355	0.006	0.64	0.1366	0.71	0.2558
600 W	0.75	0.74	0.1480	0.1499	−0.378	0.74	0.1533	−0.014	0.74	0.1505	0.82	0.2465
800 W	0.80	0.76	0.2229	0.2229	−1.002	0.77	0.2119	0.037	0.76	0.2229	0.83	0.4213

**Table 3:** Environmental impact of HD and MWHD estimated on electrical consumption and CO_2_ emission.

Distillation Threshold	Run	Experiment *q∞*	%EO ^1^	t (min) ^2^	t (h) ^2^	Distillation Rate (%EO/h)	A ^3^ per 1% EO (kWh)	E-CO_2_^4^ per 1% EO (kg)
50%	MWHD-90	0.15	0.08	37.87	0.63	0.12	0.76	0.61
	MWHD-180	0.20	0.10	5.24	0.09	1.14	0.16	0.13
	MWHD-360	0.65	0.33	5.19	0.09	3.76	0.10	0.08
	MWHD-600	0.75	0.38	4.69	0.08	4.79	0.13	0.10
	MWHD-800	0.80	0.40	3.37	0.06	7.11	0.11	0.09
	HD-205	0.55	0.28	20.50	0.34	0.80	0.25	0.20
	HD-410	0.73	0.37	6.04	0.10	3.63	0.11	0.09
85%	MWHD-90	0.15	0.13	84.24	1.40	0.09	0.99	0.79
	MWHD-180	0.20	0.17	13.43	0.22	0.76	0.24	0.19
	MWHD-360	0.65	0.55	14.53	0.24	2.28	0.16	0.13
	MWHD-600	0.75	0.64	13.19	0.22	2.90	0.21	0.17
	MWHD-800	0.80	0.68	10.14	0.17	4.02	0.20	0.16
	HD-205	0.55	0.47	65.68	1.09	0.43	0.48	0.38
	HD-410	0.73	0.62	16.89	0.28	2.20	0.19	0.15
95%	MWHD-90	0.15	0.14	105.15	1.75	0.08	1.11	0.89
	MWHD-180	0.20	0.19	18.92	0.32	0.60	0.30	0.24
	MWHD-360	0.65	0.62	24.34	0.41	1.52	0.24	0.19
	MWHD-600	0.75	0.71	21.99	0.37	1.94	0.31	0.25
	MWHD-800	0.80	0.76	30.88	0.51	1.48	0.54	0.43
	HD-205	0.55	0.52	120.00	2.00	0.26	0.78	0.63
	HD-410	0.73	0.69	28.26	0.47	1.47	0.28	0.22

^1^ Experiment *q_∞_* multiplied by Distillation Threshold percentage; ^2^ Time required for the process to reach the %EO yield; ^3^ Electrical consumption; ^4^ CO_2_ emission.

**Table 4:** Goodness of fit parameters (*R*^2^ and AARD) between SFE experimental and modeled data.

Sample	Model I		Model II	
	*R* ^2^	AARD (%)	*R* ^2^	AARD (%)
SFE-100	0.995	3.68	0.998	1.36
SFE-200	0.993	6.47	0.999	3.36
SFE-300	0.992	7.33	0.999	3.01
SFE-400	0.994	8.08	0.997	5.37
Mean	0.993	6.39	0.998	3.28

**Table 5:** Calculated parameters of two empirical models applied for SFE kinetics modeling.

Sample	Experiment	Model I		Model II				
	*Y_∞_* (%)	*Y_∞_* (%)	*k* (min^−1^)	*Y_∞_* (%)	*G*	*K_m_*	*t*_1_ (min)	*t_i_* (min)
SFE-100	2.62	2.53	0.0258	2.68	0.4176	0.2696	18.66	57.71
SFE-200	3.52	3.63	0.0166	4.12	0.4309	0.1526	34.02	106.68
SFE-300	3.62	3.64	0.0220	4.37	0.5971	0.1674	42.97	157.96
SFE-400	3.68	3.94	0.0168	3.87	0.4073	0.1636	30.00	55.31

**Table 6:** Empirical models used for HD and MWHD process modeling.

Mathematical Model	Equation	Reference
Model I	$q=q_{\infty }\left[fe^{-k_{1}t}+\left(1-f\right)e^{-k_{d1}t}\right]$	[33]
Model II	$q=q_{\infty }\left[1-\left(1-f\right)e^{-k_{d1}t}\right]$	[34]
Model III	$q=q_{\infty }\left(1-e^{-k_{d1}t}\right)$	[34]
Model IV	$q=q_{\infty }\frac{t}{\frac{1}{q_{\infty }k_{d2}}+t}$	[35]

where *k*_1_ and *k*_d1_ are the rate constants for washing and diffusion step, respectively, *k_d_*_2_ is the second-order rate constant, *q_∞_* is the asymptotic yield and *t* is distillation time (min).

## Data Availability

Not applicable.

## Sample Availability

Samples of the compounds are not available from the authors.
